# Desarrollo de la capacidad de comunicar riesgos relacionados con la exposición infantil a fluoruros, a través de una estrategia educativa en línea

**DOI:** 10.1590/0102-311XES215723

**Published:** 2024-07-22

**Authors:** Claudia Alejandra Corpus-Espinosa, Virginia Gabriela Cilia-López, Luz María Nieto-Caraveo, Ana Cristina Cubillas-Tejeda

**Affiliations:** 1 Programa Multidisciplinario de Posgrado en Ciencias Ambientales, Universidad Autónoma de San Luis Potosí, San Luis Potosí, México.; 2 Facultad de Medicina, Universidad Autónoma de San Luis Potosí, San Luis Potosí, México.; 3 Facultad de Agronomía, Universidad Autónoma de San Luis Potosí, San Luis Potosí, México.; 4 Facultad de Ciencias Químicas, Universidad Autónoma de San Luis Potosí, San Luis Potosí, México.

**Keywords:** Educación a Distancia, Educación Basada en Competencias, Educación en Salud Ambiental, Capacitación Profesional, Comunicación en Salud, Distance Education, Competency-based Education, Environmental Health Education, Professional Trainning, Health Communication, Educação a Distância, Educação Baseada em Competências, Educação em Saúde Ambiental, Capacitação Profissional, Comunicação em Saúde

## Abstract

Los fluoruros son contaminantes presentes con frecuencia y generalmente de forma natural en aguas subterráneas, y afectan a países que dependen de estas aguas para el riego y el consumo humano. La exposición crónica a fluoruros genera diversos efectos a la salud; por lo anterior, esta investigación se basó en la educación y la comunicación de riesgos para contribuir a la resolución del problema de exposición a fluoruros en la población. El objetivo fue desarrollar la capacidad de diseñar programas de comunicación de riesgos del personal involucrado en la respuesta y manejo de los riesgos ambientales para la salud, con énfasis en la exposición a fluoruros. Se diseñó e implementó un curso piloto de formación en línea sobre comunicación de riesgos y exposición a fluoruros. Para el análisis de la percepción de riesgos y conocimientos de los participantes, antes y después del curso, se aplicó un cuestionario y se llevó a cabo un grupo focal. Además, los asistentes realizaron una serie de actividades y diseñaron un programa de comunicación de riesgos con el que se valoró el grado en que se alcanzó la capacidad de desarrollar programas de comunicación de riesgos. Para mejorar el curso piloto se diseñaron y aplicaron dos encuestas de satisfacción y se realizó un análisis FODA (Fortalezas, Oportunidades, Debilidades, Amenazas). Los resultados mostraron un incremento en el nivel de conocimientos y cambios en la percepción de los participantes; en cuanto a la capacidad de diseñar programas de comunicación de riesgos, dos participantes lograron diseñarlo de manera excelente. La experiencia previa, la motivación, el compromiso para aprender y la retroalimentación brindada durante el curso, influyeron en el desarrollo de esta capacidad.

## Abstract (en)

Fluorides are contaminants that occur frequently and, generally, naturally in groundwater, affecting countries that depend on these waters for irrigation and human consumption. Chronic exposure to fluorides generates various health effects; therefore, this research was based on education and risk communication to contribute to the resolution of the problem of fluoride exposure in the population. The objective was to develop the capacity to design risk communication programs for personnel involved in the response and management of environmental health risks, with emphasis on fluoride exposure. An online pilot training course on risk communication and fluoride exposure was designed and implemented. For the analysis of the risk perception and knowledge of the participants, before and after the course, a questionnaire was applied and a focus group was conducted. In addition, the participants carried out a series of activities and designed a risk communication program to assess the degree to which the capacity to develop risk communication programs was achieved. To improve the pilot course, two satisfaction surveys were designed and implemented, and a SWOT (Strengths, Weaknesses, Opportunities, and Threats) analysis was conducted. The results showed an increase in the level of knowledge and changes in the participants’ perception; regarding the ability to design risk communication programs, two participants were able to design them excellently. Previous experience, motivation, commitment to learn and the feedback provided during the course influenced the development of this ability.

## Resumo

Os fluoretos são contaminantes que ocorrem com frequência e, geralmente, de forma natural nas águas subterrâneas, afetando os países que dependem dessas águas para irrigação e consumo humano. A exposição crônica aos fluoretos gera vários efeitos à saúde; portanto, esta pesquisa baseou-se na educação e na comunicação de riscos para contribuir com a solução do problema da exposição ao fluoreto na população. O objetivo foi desenvolver a capacidade de elaborar programas de comunicação de risco para o pessoal envolvido na resposta e no gerenciamento de riscos ambientais à saúde, com ênfase na exposição à fluoretos. Foi elaborado e implementado um curso piloto de treinamento *online* sobre comunicação de riscos e exposição. Para a análise da percepção de risco e do conhecimento dos participantes antes e depois do curso, foi aplicado um questionário e aplicado um grupo de foco. Além disso, os participantes realizaram uma série de atividades e elaboraram um programa de comunicação de riscos para avaliar até que ponto a capacidade de desenvolver programas de comunicação de riscos foi alcançada. Para aprimorar o curso piloto, foram duas pesquisas de satisfação foram desenvolvidas e implementadas e uma análise FOFA (Forças, Oportunidades, Fraquezas e Ameaças) foi aplicada. Os resultados mostraram um aumento no nível de conhecimento e mudanças nas percepções dos participantes; em termos da capacidade de elaborar programas de comunicação de riscos, dois participantes conseguiram elaborar excelentes programas de comunicação de riscos. A experiência prévia, a motivação, o compromisso com o aprendizado e o *feedback* fornecido durante o curso influenciaram o desenvolvimento dessa capacidade.

## Introducción

Los fluoruros son contaminantes presentes principalmente en aguas subterráneas, generalmente de forma natural, que afectan a países que dependen de estas aguas para el riego y el consumo, como: Argentina, Canadá, China, Estados Unidos, India, México, Pakistán, entre otros. En México, el 75% del agua suministrada a la población es subterránea, por lo que diversos estados como Chihuahua, San Luis Potosí, Guanajuato, entre otros, presentan hidrofluorosis. Además del agua, algunos dentífricos, sal, enjuagues bucales, alimentos, jugos y refrescos contribuyen a la exposición ^1^ ^,^ ^2^ ^,^ ^3^. La exposición crónica se manifiesta principalmente por fluorosis dental y esquelética, pero existen otros efectos a la salud ^4^. Los fluoruros son un reto importante para la salud pública en el mundo ^5^ ^,^ ^6^, por tanto, es necesario implementar estrategias para disminuir la exposición, principalmente en la población infantil de 0 a 12 años de edad, al ser especialmente susceptibles porque están en desarrollo ^4^ ^,^ ^7^.

La exposición a diversos peligros ha generado el desarrollo de investigación, experiencias y conocimientos sobre la eficacia de la comunicación de riesgos como estrategia comunitaria para mitigar riesgos, entre ellos la exposición al agua contaminada ^8^ ^,^ ^9^. La comunicación de riesgos ha evolucionado a medida que sus componentes (intencionalidad, contenido, audiencia blanco, fuente y flujo de los mensajes) han sido interpretados ^10^ ^,^ ^11^. En un inicio, se consideraba como un proceso que implicaba la transferencia de hechos científicos relacionados con un riesgo y un conjunto de conclusiones de los expertos hacia el público lego. Sin embargo, se reconoció que los expertos no son los únicos gestores de la información, por lo que se debe tomar en cuenta los aportes culturales, conocimientos y experiencias de las personas, es decir, su percepción de riesgos, la cual es el conjunto de juicios subjetivos sobre la probabilidad de eventos negativos, y se construye a nivel social, reflejando valores, símbolos, ideología e historia ^11^ ^,^ ^12^. La experiencia, la información y los antecedentes culturales forman una tríada inseparable que da forma a la percepción de riesgos, aunque estas no son las únicas variables relacionadas ^13^.

La Organización Panamericana de la Salud (OPS) ^14^ define la comunicación de riesgos como “*el intercambio en tiempo real, de información, recomendaciones y opiniones, entre expertos o funcionarios y personas que se enfrentan a una amenaza (riesgo) para su sobrevivencia, su salud o su bienestar económico o social. El objetivo es que toda persona expuesta a un riesgo sea capaz de tomar decisiones informadas para mitigar los efectos de la amenaza (riesgo), como el brote de una enfermedad, y tomar las medidas y acciones de protección y prevención*”. Diversos estudios señalan que los peligros que les preocupan a las personas y cómo se enfrentan y comportan ante ellos se determinan por su percepción de riesgos, por lo que conocerla permite desarrollar intervenciones para que el público sea capaz de percibir los riesgos con precisión y tomar decisiones adecuadas al respecto. Es así como la percepción del riesgo y la comunicación de riesgo se vinculan mediante relaciones recíprocas ^13^ ^,^ ^15^ ^,^ ^16^.

De acuerdo con la Organización Mundial de la Salud (OMS), es necesaria la constante preparación y fortalecimiento de las capacidades de todos los involucrados en la respuesta y en el manejo de los riesgos, para que sean capaces de prevenirlos o responder apropiadamente y, debido a la importancia de la comunicación de riesgos como intervención para la protección de la salud, se exige la elaboración y evaluación de capacidades como parte del manejo del riesgo ^17^. Por lo que es importante buscar estrategias para la formación y profesionalización de la comunicación de riesgos. En este sentido, es fundamental la formación del personal que labora en las diferentes instituciones, tanto públicas como privadas, involucradas en la mejora de la salud de la población; tanto para promover el uso de información científica, como para desarrollar intervenciones para reducir riesgos a la salud. Desafortunadamente, es frecuente que exista una falta de vinculación entre investigadores, tomadores de decisiones y el personal involucrado en la respuesta y manejo de riesgos ambientales ^18^. Este estudio es un ejemplo de estrategia para la vinculación.

Actualmente, es posible generar, compartir y comunicar información y conocimiento, así como desaparecer las barreras espaciotemporales gracias a las tecnologías de la información y comunicación (TIC). En este sentido, la educación en línea se ha convertido en una modalidad de formación, que facilita la participación activa en el aprendizaje en cualquier momento y lugar ^19^ ^,^ ^20^ ^,^ ^21^. La OPS ^22^ (p. 12) señala que “*se deben diseñar, adaptar y reorientar las propuestas educativas, por medio de una combinación pertinente de la pedagogía y la tecnología disponibles*”.

Con base en lo anterior, el objetivo del presente trabajo fue desarrollar la capacidad de diseñar programas de comunicación de riesgos del personal involucrado en el manejo y respuesta a riesgos ambientales para la salud, con énfasis en la exposición infantil a fluoruros en las regiones de San Luis Potosí y de Guanajuato, México, mediante la educación en línea.

## Metodología

Se desarrolló una investigación bajo un enfoque mixto y de triangulación para hacer un cruce de la información recabada y que esta tenga mayor validez ^23^ ^,^ ^24^ ^,^ ^25^.

### Curso

Se diseñó un curso piloto de formación en línea cuyo objetivo fue desarrollar en los participantes conocimientos y habilidades que les permitieran diseñar programas de comunicación de riesgos para abordar amenazas a la salud, entre ellas, la exposición a fluoruros, sus efectos en la salud y las medidas adecuadas de prevención.

El curso duró 47 horas, 30 síncronas y 17 asíncronas, con un enfoque dirigido y autodirigido; constó de 10 temas, una introducción y una evaluación. Las sesiones fueron los sábados de cada semana (con duración de dos horas), del 16 de enero al 8 de mayo de 2021; las plataformas utilizadas fueron Zoom (https://zoom.us/) y Google Classroom (https://classroom.google.com/). El curso fue avalado por la Secretaría Académica de la Universidad Autónoma de San Luis Potosí; a quienes lo concluyeron satisfactoriamente se les entregó una constancia con valor curricular.

### Participantes

El curso se dirigió al personal involucrado en el manejo y respuesta a riesgos ambientales, de la Comisión Estatal para la Protección contra Riesgos Sanitarios (COEPRIS) y del Sistema para el Desarrollo Integral de las Familias (DIF), de San Luis Potosí y de Guanajuato; instituciones con las cuales ya se tenía una vinculación previa. La invitación se realizó por un cartel digital enviado por correo electrónico a las autoridades correspondientes de cada institución, quienes difundieron la invitación entre su personal.

Como criterio de inclusión se pidió a los participantes que sus actividades laborales se relacionaran con la gestión y comunicación de riesgos en entornos laborales o comunitarios. Se inscribieron 11 personas de manera voluntaria.

### Evaluación de los participantes

Previo al inicio del curso, se analizó en los participantes sus conocimientos y habilidades para el diseño de programas de comunicación de riesgos, así como su conocimiento y percepción de riesgos sobre la exposición a fluoruros. Con tal fin se aplicó un cuestionario en línea a través de Microsoft Forms (https://forms.office.com/), que constó de preguntas abiertas y cerradas. El mismo cuestionario se aplicó al finalizar el curso con la finalidad de determinar cambios.

Las preguntas abiertas para evaluar la percepción de riesgos se sometieron a análisis de contenido ^26^ ^,^ ^27^, por lo que se establecieron categorías temáticas con base en las respuestas dadas y posteriormente se clasificaron y obtuvieron frecuencias y porcentajes de los participantes que respondieron en cada una de las categorías. Para las preguntas cerradas se obtuvo la frecuencia de las personas que respondieron en cada una de las opciones. Las preguntas para analizar conocimientos se calificaron como correctas o incorrectas mediante criterios previamente establecidos basados en fuentes bibliográficas, lo que permitió obtener una nota final (escala de 0 a 10).

La preevaluación se realizó el 16 de enero de 2021 y se evaluó a los 11 participantes; los resultados obtenidos permitieron realizar adecuaciones al curso antes de impartirlo, para que estuviera adaptado a las personas y a su contexto. Después del curso, seis participantes (quienes lo concluyeron) contestaron el cuestionario el 8 de mayo de 2021. Además del cuestionario, para evaluar la percepción de riesgos, se desarrolló un grupo focal antes del curso y otro al finalizarlo. Las respuestas se analizaron mediante análisis del discurso ^28^ ^,^ ^29^.

Para evaluar la capacidad de diseñar programas de comunicación de riesgos, los participantes elaboraron una propuesta, y durante el curso se les brindó retroalimentación en sus avances. La evaluación de la propuesta se realizó mediante una rúbrica (niveles de logro: excelente, regular, bajo) que contempló once rubros, ocho de los cuales corresponden a las etapas para el diseño de un programa de comunicación de riesgos. Aunado a lo anterior, se realizaron actividades que consistieron en: (a) la elaboración de mapas conceptuales; (b) el análisis de los pasos de un programa de comunicación de riesgos; (c) el diseño de una infografía y un mapa mental sobre la exposición a fluoruros, (d) la identificación de fortalezas y debilidades de ejemplos de programas de comunicación de riesgos. Dichas actividades fueron evaluadas a través de rúbricas de evaluación diseñadas por las autoras (niveles de logro: excelente, regular, bajo). La calificación final se obtuvo con base en la nota obtenida en el cuestionario (exclusivamente las preguntas sobre conocimientos), el promedio de las actividades entregadas y la calificación obtenida en la propuesta del programa de comunicación de riesgos, los cuales tuvieron un valor del 20%, 20% y 60% respectivamente del total de la calificación.

Para mejorar el curso se diseñaron y aplicaron dos encuestas de satisfacción y se hizo un análisis FODA (Fortalezas, Oportunidades, Debilidades, Amenazas). Una de las encuestas se aplicó a mitad del curso para hacer ajustes a las sesiones restantes, basados en las respuestas obtenidas, y la otra al final; ambas tuvieron preguntas abiertas y de escala Likert clasificadas en: generalidades del curso, organización, contenido, estrategias de aprendizaje, evaluación, instructoras, plataformas, autoevaluación y comentarios generales. El análisis FODA, es útil para la evaluación de cursos y programas y su posterior mejora, potenciando las fortalezas y oportunidades y minimizando las debilidades y amenazas ^30^ ^,^ ^31^. Es imperativo dejar claro que no se realizó una evaluación del curso en sí mismo, sino más bien se evaluó la satisfacción de los participantes con el curso, así como las fortalezas, oportunidades, debilidades y amenazas.

### Análisis estadístico

Para analizar al aprendizaje logrado, se hicieron comparaciones entre las notas obtenidas en la evaluación previa y posterior al curso, para lo cual se utilizó la prueba no paramétrica U de Mann-Whitney para comparar los promedios grupales antes y después de la implementación del curso, así como la prueba de Wilcoxon para comparar las notas individuales. Se utilizó la prueba de normalidad de Shapiro-Wilk, y el programa estadístico R versión 4.1.2 (http://www.r-project.org).

### Consideraciones éticas

Esta investigación formó parte del proyecto *Evaluación de la Exposición a Flúor, Ftalatos y Microplásticos en Bebidas de Consumo Infantil* que fue aprobado por el Comité de Ética en Investigación de la Facultad de Medicina, Universidad Autónoma de San Luis Potosí (número de registro: CEI-2019-003).

## Resultados

En la Tabla 1 se presentan las características de los participantes, quienes reportaron tener los siguientes empleos: promotores sanitarios, orientadores alimentarios, verificadores sanitarios, coordinadores de evidencia de riesgos y estudiantes. También mencionaron ser licenciados en informática, ingenieros ambientales, médicos veterinarios zootecnistas, nutriólogos e ingenieros químicos.

**Tabla 1:** Características de los participantes del curso.

Características	Participantes al inicio (n = 11)		Participantes al final (n = 6)	
	%	Frecuencia	%	Frecuencia
Sexo				
Masculino	55	6	33	2
Femenino	45	5	67	4
Lugar de procedencia				
Guanajuato	27	3	50	3
San Luis Potosí	73	8	50	3
Escolaridad				
Licenciatura	82	9	67	4
Maestría	18	2	33	2
Situación laboral				
Trabajador de COEPRIS (San Luis Potosí)	55	6	33	2
Trabajador en DIF (Guanajuato)	27	3	50	3
Ex alumna de la UASLP	18	2	17	1

COEPRIS: Comisión Estatal para la Protección contra Riesgos Sanitarios; DIF: Sistema para el Desarrollo Integral de las Familias; UASLP: Universidad Autónoma de San Luis Potosí.

### Percepción de riesgos

Con base en los resultados de la pre y post evaluación y de los grupos focales, se observó que antes de tomar el curso, el 27% (n = 3) de los 11 participantes consideró el agua insalubre como un elemento que puede dañar su salud, refiriendo principalmente la contaminación biológica y física, y en menor medida la química. En la evaluación posterior al curso, el 100% (n = 6) de los participantes percibió el agua insalubre como un riesgo para la salud, mencionando la contaminación química específicamente con fluoruros.

Con relación al consumo de agua, al comparar a únicamente a los seis participantes que concluyeron el curso, se observó que en la preevaluación el 83% (n = 5) refirió utilizar agua purificada para beber, y después del curso el 100% (n = 6). Con respecto al agua utilizada para preparar alimentos, antes del curso el 33% (n = 2) refirió utilizar agua purificada, al concluir el curso, el 83,3% (n = 5). Es importante resaltar que, en el grupo focal realizado después del curso, una de las personas indicó implementar cambio de hábitos en su familia con respecto a esta práctica: “*Tuve la oportunidad de revisar la página que nos compartías de la escuela más cercana aquí de mi casa y si también hay presencia de flúor en el agua, entonces en mi casa… mi mamá si utilizaba una parte del agua de la cisterna para hacer la cocción de frijoles y eso pues ya es una práctica que hemos omitido*”.

**Figura 1:**
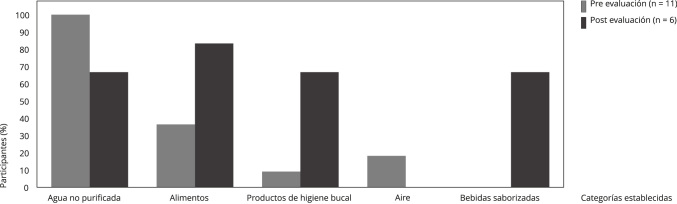
Fuentes de posible exposición a fluoruros referidas por los participantes, antes y después del curso en línea, enero y mayo de 2021.

Con respecto a las fuentes de exposición (Figura 1), en la preevaluación todos refirieron como principal fuente el consumo de agua no purificada; en la postevaluación, la mayoría también incluyó alimentos, bebidas saborizadas y productos de higiene bucal. Con relación a los efectos sobre la salud, antes del curso, los participantes mencionaron la fluorosis dental y esquelética y desconocían si afectaba más a adultos o a la población infantil. Por el contrario, después del curso todos coincidieron en que los niños y niñas tienen un mayor riesgo. Además, mencionaron una mayor variedad de efectos como daño renal y neurológico.

**Figura 2:**
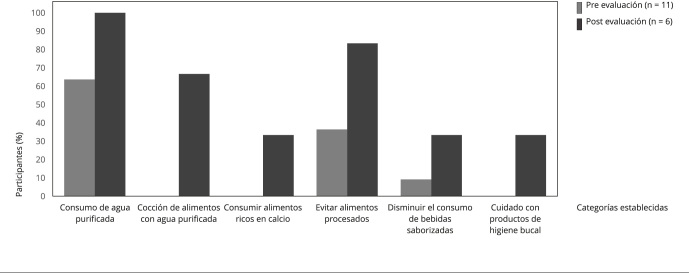
Medidas de prevención de la exposición a fluoruros referidas por los participantes, antes y después del curso en línea, enero y mayo de 2021.

En cuanto a las medidas de prevención, después del curso todos los participantes reportaron el consumir agua purificada como una de las medidas preventivas, y se encontró un aumento en los participantes que refirieron otras medidas, además de hacer énfasis en el cuidado de la población infantil (Figura 2). Con respecto a las estrategias de intervención que conocían para disminuir la exposición a fluoruros, antes del curso ninguno de los participantes mencionó la comunicación de riesgos como una estrategia, y aunque uno de ellos mencionó haber sido parte de un programa de comunicación de riesgos, se refirió a este como una actividad para brindar información. Sin embargo, después del curso las personas reconocieron a la comunicación de riesgos como una estrategia de intervención útil para disminuir la exposición a fluoruros, concibiéndola, además, como un proceso bidireccional, haciendo énfasis en la importancia de la participación comunitaria y en la conformación de equipos multidisciplinarios.

Al finalizar el curso, también se encontró que todos percibieron que la presencia de fluoruros en el agua en los estados de México en los que viven es una gran problemática y propusieron estrategias de comunicación de riesgos para implementar en las comunidades con las que trabajan. Otro dato relevante es que evidenciaron la falta de vinculación entre la academia y el personal que, como ellos, trabajan en instituciones que manejan y dan respuesta a riesgos para la salud: “*Nosotros damos orientación alimentaria y… nosotros diciendo hierve el agua para beber o para los alimentos* (…) *pues es algo que no conocíamos…, entonces cuando demos la orientación lo podríamos comentar* (...) *a mí se me ocurre trabajar con mamás que ya tenemos un poquito más de contacto, pero yo creo que sí es muy importante que tanto Secretaría de Educación como Secretaría de Salud estén involucradas en un proyecto así*”.

### Evaluación de conocimientos

En la preevaluación, la media de la calificación obtenida por los participantes (n = 11) fue 2,8 (desviación estándar - DE: ± 0,97), y en la postevaluación la media de la calificación obtenida por los participantes (n = 6) incrementó de manera estadísticamente significativa a 8,2 ± 1,65 (p = 0,00016). Por otro lado, al comparar las calificaciones individuales (Tabla 2), se observó que hubo un incremento significativo en la calificación obtenida por cada participante (p = 0,03125). Después del curso, la integración de conocimientos se observó principalmente en el tema de comunicación de riesgos. Respecto al tema de los fluoruros, la mayoría de los participantes tenía conocimientos previos, sin embargo, en la evaluación posterior al curso, se observaron diferencias en la forma de redacción de sus respuestas, ya que fueron más completas e integraron conceptos revisados.

**Tabla 2:** Calificación obtenida por participante en el cuestionario para evaluar conocimientos.

Participantes	Antes del curso	Después del curso
Participante 1	3,5	8,6
Participante 2	4,7	8,8
Participante 3	3,7	9,3
Participante 4	3,0	9,5
Participante 5	2,0	5,0
Participante 6	2,9	8,2
Media	3,3	8,2
Desviación estándar	± 0,91	± 1,65

En cuanto a las actividades adicionales, se desarrollaron 15, de las cuales seis fueron calificadas a lo largo del curso y nueve correspondieron al diseño del programa de comunicación de riesgos. De los seis participantes que finalizaron el curso, solo tres completaron todas las actividades y con base en los niveles de logro, la mayoría obtuvo niveles excelentes.

### Propuesta de un programa de comunicación de riesgos

La actividad medular del curso fue el diseño de una propuesta de programas de comunicación de riesgos; de los seis participantes que lo concluyeron, cinco entregaron su propuesta. En la Tabla 3 se muestran los niveles de logro alcanzados por los participantes en cada uno de los criterios evaluados. En el criterio “determinación de la problemática de salud ambiental”, el 60% de los participantes obtuvo un nivel excelente, lo que implicó que fueron capaces de analizar la situación ambiental en la que decidieron trabajar, analizando sus causas y las consecuencias a la salud humana que se derivan de la exposición al riesgo. Para realizarlo se apoyaron en información obtenida a través de uso fuentes indirectas, es decir, que recurrieron a bases de datos, libros, ensayos, entre otras fuentes ^32^. En el desarrollo de este criterio, el 20% alcanzó un nivel de logro regular, ya que le hizo falta plasmar el riesgo a la salud humana derivado del problema que decidió abordar; el 20% obtuvo un nivel de logro bajo porque no presentó la problemática de salud ambiental que abordaría desde la comunicación de riesgo.

**Tabla 3:** Niveles de logro obtenido en los criterios de evaluación, por los cinco participantes que entregaron su propuesta.

Criterio evaluado	Nivel excelente		Nivel regular		Nivel bajo	
	%	n	%	n	%	n
Contenido	60	3	0	0	40	2
Determinación de la problemática de salud ambiental	60	3	20	1	20	1
Contextualización	100	5	0	0	0	0
Definición y análisis de la audiencia blanco	80	4	20	1	0	0
Establecimiento de objetivos	60	3	20	1	20	1
Establecimiento del equipo de comunicación de riesgos	100	5	0	0	0	0
Diseño de las estrategias y elaboración de mensajes	40	2	40	2	20	1
Puesta en operación del programa	60	3	20	1	20	1
Evaluación	40	2	40	2	20	1
Escritura y presentación	20	1	60	3	20	1
Fuentes bibliográficas	40	2	40	2	20	1

Por otra parte, en el criterio de “contextualización”, todos los participantes contemplaron aspectos sociales, ambientales, económicos y culturales al momento de describir el sitio en donde plantearon realizar el programa, esta descripción fue hecha a través de la consulta de fuentes secundarias.

Respecto al criterio “definición y análisis de las audiencias blanco”, se encontró que el 80% de los participantes logró identificar la población a la que estaría dirigido su programa; además plantearon diversas herramientas, tanto grupales como individuales, para el análisis de la audiencia con el propósito de evaluar su percepción de riesgos, conocimientos y necesidades; sin embargo, el 20% no identificó la población objetivo ni propuso herramientas para evaluarla. En lo que se refiere al criterio de “establecimiento de objetivos”, el 60% de los participantes fue capaz de plantear objetivos, tanto el general como los específicos, de manera medible, alcanzable y realista.

En el criterio de “establecimiento del equipo de comunicación de riesgo”, todos lograron seleccionar un grupo multidisciplinario y propusieron instituciones con las que se podrían vincular para el desarrollo de su programa. Respecto al “diseño de las estrategias y elaboración de mensajes”, el 40% de los participantes diseñó mapas de mensaje, en donde los mensajes clave fueron claros, en voz activa, cortos y cubrieron todas las rutas de exposición. Además, plantearon una campaña de medios de comunicación, tanto para población adulta como infantil, y diseñaron uno de los medios de comunicación propuestos. Quienes obtuvieron un nivel de logro regular, la principal dificultad que presentaron fue el diseño de los mensajes clave, ya que estos no cubrían todas las rutas de exposición a fluoruros y fueron poco específicos. Respecto a los medios de comunicación, a pesar de mencionarlos, no los describieron ni justificaron su uso, además de no diseñar el medio de comunicación solicitado. Quien obtuvo un nivel de logro bajo fue porque no presentó ninguna estrategia.

En el criterio “puesta en operación del programa”, se solicitó que calcularan el presupuesto necesario para su desarrollo, así como la planificación del cronograma de actividades y que incluyeran de qué manera involucrarían a la comunidad en el programa. Con base en lo anterior, se encontró que el 60% de los participantes obtuvo un nivel excelente, el 20% un nivel regular porque no especificó cómo involucraría a la comunidad y quien alcanzó el nivel bajo fue porque no desarrollo este apartado.

Finalmente, se analizó cómo proponían evaluar el programa; en este criterio el 40% de los participantes plantearon los tres tipos de evaluación revisada en el curso: formativa, de proceso y de resultado. El 40% logró un nivel regular porque les faltó mencionar alguno de los tipos de evaluación y el 20 % obtuvo un nivel bajo porque no presentó la forma de evaluación.

### Encuestas de satisfacción y análisis FODA

Se encontró un alto nivel de satisfacción sobre la temática abordada, la organización del curso, las estrategias de aprendizaje, la retroalimentación brindada, la forma de evaluación, entre otros aspectos. Además, los participantes mencionaron que los contenidos fueron valiosos para su formación profesional y personal; sin embargo, hubo ciertos obstáculos que les dificultaron el desarrollo del curso, tales como el acceso a una buena conexión de internet, el acceso a dispositivos como computadora, tableta o teléfono celular. Asimismo, algunas personas reportaron problemas para comprender algunos de contenidos, derivado de la falta de conocimientos previos en el área, y más de la mitad consideraron que la carga de trabajo por semana fue elevada.

Dentro de las fortalezas y oportunidades del curso, señalaron el uso de la comunicación tanto asíncrona como síncrona, ya que facilitó la participación y el mantenimiento del interés y existió la posibilidad de generar alianzas con profesionales e instituciones que realizan trabajo comunitario. Respecto a las debilidades y oportunidades, se mencionaron el poco tiempo para el desarrollo de las actividades, fallas en la conexión de internet, la carga laboral y situaciones personales, lo que retrasó o dificultó la entrega de las actividades.

Finalmente, con base en los resultados anteriormente expuestos, se rediseñó el curso para desarrollar la capacidad de diseñar programas de comunicación de riesgos que fue el principal aporte y el producto final de esta investigación.

## Discusión

La propuesta aquí presentada se sustenta en la educación y la comunicación de riesgos para contribuir a la resolución del problema de exposición a fluoruros; asimismo, hace una contribución a la investigación sobre la educación en línea como medio para la formación y desarrollo de capacidades del personal involucrado en la respuesta y manejo de los riesgos ambientales para la salud.

Los resultados obtenidos mostraron que los participantes del curso aumentaron su calificación independientemente del nivel educativo o experiencia previa; estos hallazgos sugieren que este curso podría ser una herramienta efectiva para mejorar el conocimiento. Lo anterior coincide con investigaciones realizadas por van de Steeg et al. ^33^ y Salter et al. ^34^, quienes concluyen que el aprendizaje en línea logra incrementar conocimientos; sin embargo, hay evidencia limitada de que mejore capacidades o la práctica profesional. Asimismo, se observaron modificaciones en la percepción de riesgos de los participantes sobre la exposición a fluoruros. Es importante señalar que tanto los conocimientos como la percepción fueron analizados una semana después de concluido el curso; por lo que sería relevante evaluar su sostenibilidad a largo plazo.

Con respecto a la capacidad de diseñar un programa de comunicación de riesgos, dos participantes lograron diseñar de manera excelente la estrategia del programa y dos de manera regular, por lo que se puede concluir que el curso piloto contribuyó al desarrollo de esta capacidad. La experiencia previa en la temática, la motivación que tienen para aprender, la retroalimentación brindada a lo largo del curso y el compromiso de cada participante, jugaron un papel importante en la obtención de un nivel competente. Estos resultados están en consonancia con lo reportado en los estudios de Vaz-Fernandes & Caeiro ^35^, y González-Soto & Farnós-Miró ^36^.

Los resultados obtenidos mostraron que cuando se solicitó el desarrollo de etapas del proceso de comunicación de riesgos que resultaban más sencillas, porque consistían principalmente en la búsqueda de información, como lo fue el planteamiento del contexto, la determinación de la problemática de salud ambiental, la definición de la audiencia blanco y el establecimiento del equipo de comunicación de riesgos; se observó que un mayor número de participantes logró niveles de desempeño excelente y en menor medida un nivel regular. Sin embargo, cuando se solicitó el diseño de etapas más complejas, es decir, que requerían además de la búsqueda de información la puesta en práctica de lo aprendido, como lo fue el establecimiento de objetivos, la estrategia de comunicación, la selección de medios de comunicación, la elaboración de mensajes clave, así como el planteamiento de la evaluación del programa, la mayoría de los participantes logró niveles entre regular y bajo.

Tomando en cuenta la experiencia previa en el tema, los participantes que reportaron que habían sido parte de algún programa de comunicación de riesgos, tomaron un curso o su labor profesional involucra el diseño y gestión de programas comunitarios, alcanzaron principalmente niveles de logro excelentes en los rubros evaluados, en especial en el criterio del diseño de las estrategias, de los mensajes clave y en la evaluación. Este resultado coincide con una de las preguntas realizadas en la encuesta de satisfacción, la cual buscaba conocer qué conocimientos previos consideraban útiles los participantes para no tener dificultades con los contenidos del curso. Aquellas personas que consiguieron principalmente el nivel excelente y que tenían experiencia previa, reportaron no necesitar ningún conocimiento previo o únicamente requerir orientación en la búsqueda de información en fuentes confiables y referencias bibliográficas. Pero los que mencionaron que les hubiera sido útil tener conocimientos sobre salud ambiental y antecedentes sobre el diseño de programas de comunicación de riesgos, obtuvieron en la mayoría de los rubros evaluados, niveles regulares o bajos, especialmente en los criterios del diseño de la estrategia y la evaluación.

En lo que se refiere a la retroalimentación, se observó que los participantes que entregaron avances de sus propuestas de programa a lo largo del curso obtuvieron un mejor nivel de logro en comparación con los que en ocasiones no los entregaron. En este sentido, la retroalimentación brindada les permitió conocer cuáles eran sus errores y mejorarlos en la entrega final, tal como lo señalan también Vaz-Fernandes & Caeiro ^35^. Además, lo anterior coincide con lo reportado en la encuesta de satisfacción, en donde los participantes mencionaron estar satisfechos con la retroalimentación que se les dio. Aunque es necesario mencionar que como parte de la retroalimentación es indispensable hacer más énfasis en la importancia de la participación comunitaria y cómo fomentarla durante un programa de comunicación de riesgos.

Sobre la motivación para tomar el curso, aquellos participantes cuya motivación fue la aplicación de los aprendizajes adquiridos en su entorno laboral, especialmente en el desarrollo de programas comunitarios, cuando diseñaron la propuesta de programa obtenían resultados más exitosos, en especial en el diseño de las estrategias, la elaboración de los mensajes clave y la evaluación, en comparación con aquellos que mencionaron que su motivación era la adquisición de nuevos conocimientos o la constancia con valor curricular. Estos datos coinciden con lo reportado por Lee et al. ^37^ quienes mencionan que, en la educación en línea, el compromiso y la motivación son esenciales para que exista un aprendizaje efectivo y significativo.

Durante la pandemia de COVID-19, hubo un incremento en el agotamiento de las personas debido al aumento en las horas laborales, la necesidad de conciliarlas con asuntos personales, entre otros factores ^38^ ^,^ ^39^ ^,^ ^40^. Esta relación entre agotamiento y educación en línea ha sido objeto de debate y reflexión. Aunque la educación en línea ofrece ventajas como la flexibilidad de horarios y la accesibilidad desde cualquier ubicación ^22^, también presenta desafíos, como la falta de límites claros entre el trabajo y la vida personal. En este contexto, el curso piloto podría haber representado una carga adicional para los participantes, ya que, quienes no concluyeron el curso, refirieron la sobrecarga laboral y cuestiones personales. Además, las TIC pueden plantear desafíos y frustraciones, especialmente para quienes no están familiarizados con ellas. Por ejemplo, problemas con la conexión a internet o la falta de acceso a dispositivos para conectarse fueron mencionados por los participantes, lo que pudo aumentar la sensación de agotamiento y desmotivación. En la actualidad, diversas instituciones emplean la educación en línea para formar personas que trabajan a tiempo completo. Por tanto, es crucial considerar estos aspectos en el diseño y desarrollo de los cursos, para evitar que representen una carga adicional y se comprometa la salud física o mental.

Al momento de desarrollar la matriz FODA se identificó que no se ofertaban cursos sobre el desarrollo de la capacidad para diseñar programas de comunicación de riesgos en México, o sobre la exposición a fluoruros, por lo que es importante seguir ofertándolos. Pero, se sugiere en futuros cursos, fomentar la colaboración entre diversos actores clave, como la academia, el personal encargado de la gestión de riesgos ambientales para la salud de entidades gubernamentales o empresas, e incluso organizaciones de la sociedad civil, con el propósito de aumentar el impacto social de estas iniciativas, además de contribuir a su sostenibilidad ^41^. Además, se debe considerar la evaluación de la efectividad en la formación del personal involucrado en la gestión de riesgos ambientales para la salud, enfatizando el desarrollo de capacidades, más que la simple retención de contenidos ^42^.

Este estudio tiene limitaciones, entre ellas la capacidad de inferencia únicamente a la población seleccionada, es decir, los participantes del curso. No obstante, puede servir como modelo para el diseño de otros cursos. Asimismo, no se controlaron factores de confusión ni se realizaron pruebas del tamaño del efecto, lo que impide atribuir completamente los cambios en conocimientos y percepciones únicamente al curso. Otra limitante fue la baja participación en el curso por parte del personal de las instituciones invitadas, debido, quizás, a la situación ocasionada por la pandemia de COVID-19, o bien, por falta de promoción. Para futuros cursos se sugiere ampliar el período de difusión y promocionarlo acorde al público objetivo.
